# Supplementary material for: Predictive Biomarkers for Checkpoint Inhibitor Immune-Related Adverse Events
Source: Cancers (Basel). 2023 Mar 6;15(5):1629. doi: 10.3390/cancers15051629 (PMC10000735; doi:10.3390/cancers15051629)
Supplement: Supplementary file 1 [file cancers-15-01629-s001.zip › cancers-2220161-supplementary.pdf]

Materials and Methods

Among the different types of reviews [226], we chose to conduct a literature review to provide an overview of the state of the art in the field of predictive irAE biomarkers.

We included a range of different study designs, namely, retrospective, prospective, case-control, cohort studies, clinical trials, and case series including more than five cases, and studies conducted in either animals or humans. Previous review articles were not explicitly included in this review, although they were used for identifying original studies missed in the electronic search. The search was restricted to articles written in English.

As recommended in the PRISMA 2020 statement for reporting systematic reviews [227], a flow diagram was constructed indicating the references corresponding to each phase: identification, screening, and inclusion (Supplementary Figure S1). In the identification phase, we applied a two-step strategy starting with a generic PubMed literature search for related publications without time limitation using the terms "immune-related adverse events" and "predictive biomarkers" as keywords. In a second step, we proceeded to iterative specific searches using the term "immune-related adverse events" and the corresponding MeSH term for each biomarker under review. For instance, a specific search was performed by combining the term "immune-related adverse events" with the MeSH term "autoantibodies", and so on, for all other potential predictive factors of irAEs included in this review; namely "autoantigens", "blood cell count" and ratios, "cytokines", "blood proteins" and other biological fluid "proteins", "HLA antigens", "micro-RNAs", "genetic variations" and "gene expression profiling", and "gastrointestinal microbiome".

After removing duplicate PubMed entries, references were screened by their title and abstract, excluding any records that did not address the topic under review. For the full-text analysis of the selected publications including their reference lists, snowballing was used to search for other related articles not detected in the two preliminary identification steps. Case series including five patients or fewer and articles on antitumor efficacy rather than toxicity were excluded. In the event of doubts, the final decision to include a particular publication was made by consensus. Finally, 204 articles were included in the review.

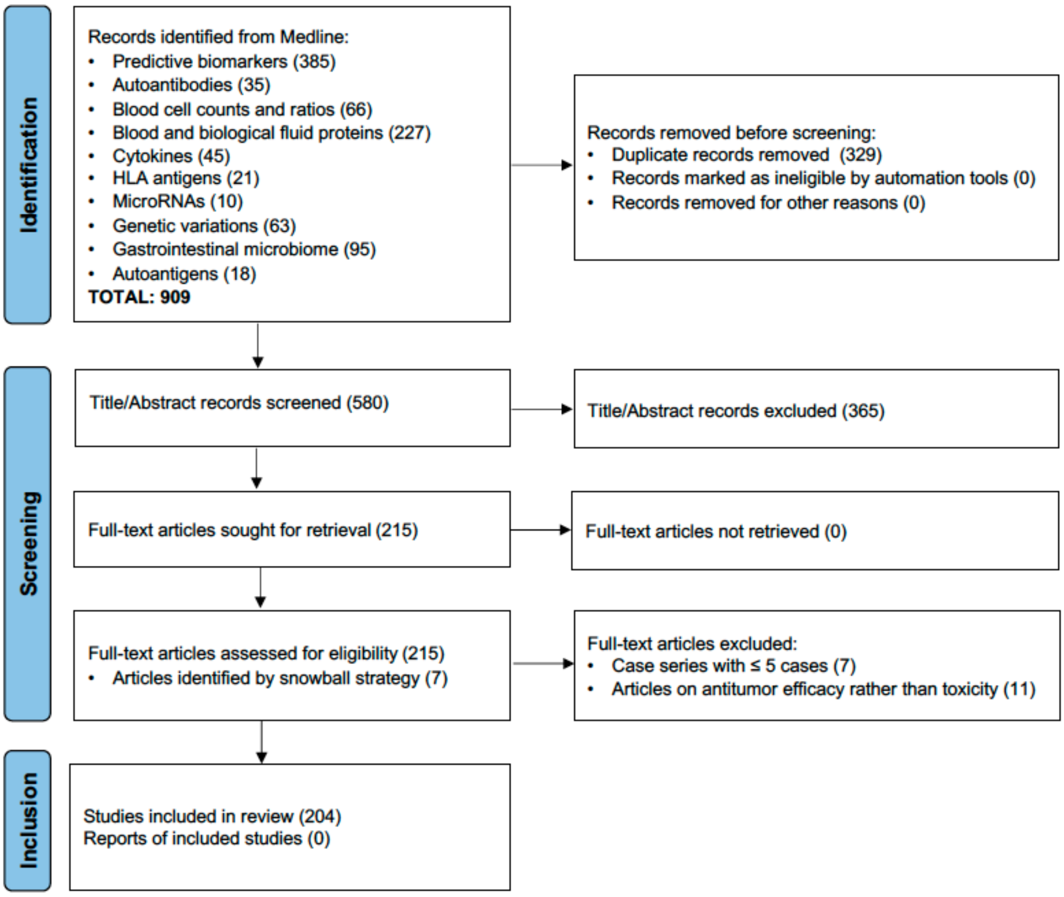

Supplementary Figure S1. Diagram flow of studies identified via PubMed, screened, and finally included in the review, based on the PRISMA 2020 statement.
